# Monoclonal antibody 1B9 against the African swine fever virus viroporin pB169L: molecular characterization and epitope mapping

**DOI:** 10.3389/fmicb.2026.1942750

**Published:** 2026-09-22

**Authors:** Zhiying Cui, Jianing Feng, Miaomiao Ye, Qin Wen, Peng Gao, Qiongqiong Zhou, Yongning Zhang, Lei Zhou, Xinna Ge, Xin Guo, Jun Han, Hanchun Yang

**Affiliations:** 1 State Key Laboratory of Veterinary Public Health and Safety, College of Veterinary Medicine, China Agricultural University, Beijing, China; 2 Key Laboratory of Animal Epidemiology, Ministry of Agriculture and Rural Affairs, College of Veterinary Medicine, China Agricultural University, Beijing, China

**Keywords:** African swine fever virus, monoclonal antibody, pB169L, viral factory, viroporin

## Abstract

**Introduction:**

The African swine fever virus (ASFV) viroporin pB169L is a conserved membrane protein, but well-defined reagents for its detection and molecular characterization remain limited.

**Methods:**

A soluble recombinant antigen comprising the non-transmembrane regions of pB169L was used to generate a mouse monoclonal antibody (mAb), designated 1B9. The antibody specificity was characterized by immunoblotting and immunofluorescence assays. The expression and subcellular localization of pB169L were examined in ASFV-infected WSL-R4 cells and primary porcine alveolar macrophages. The variable regions of 1B9 were sequenced, and its epitope was mapped using truncated antigenic fragments.

**Results:**

The 1B9 antibody belonged to the IgG1 kappa subclass and recognized pB169L by immunoblotting and immunofluorescence at a working dilution of 1:2,000. In ASFV-infected cells, pB169L was detectable by immunofluorescence from 6 h post-infection and by Western blot from 12 h post-infection, with progressive accumulation in cytoplasmic viral factories together with p54. Two immunoreactive species of approximately 18 and 21 kDa were consistently observed. Variable-region sequencing defined the VH/VL gene usage and complementarity-determining regions of 1B9. Epitope mapping identified a linear epitope at residues ^147^SKPLVPLQE^155^, which was broadly conserved across several ASFV genotypes and predicted to reside in a hydrophilic, flexible, surface-exposed C-terminal region.

**Discussion:**

Thus, 1B9 provides a useful reagent for studying the expression, localization, and antigenic properties of pB169L.

## Highlights

- Monoclonal antibody 1B9 specifically detects ASFV pB169L by immunoblotting and infected-cell imaging.
- The linear epitope maps to pB169L residues 147–155 (SKPLVPLQE).
- VH and VL sequences support recombinant antibody engineering.

## 1 Introduction

African swine fever (ASF) is a highly lethal hemorrhagic disease caused by African swine fever virus (ASFV) and poses a major threat to the global swine industry (Wang et al., 2019; Fuchs et al., 2025; Yang et al., 2025). Since its first report in 1921, ASFV has been classified into 25 genotypes on the basis of the B646L gene (Montgomery, 1921; Li et al., 2026). Genotypes I and II are notable for intercontinental spread: genotype I reached Europe and South America in 1957, whereas genotype II emerged in the Caucasus in 2007 and spread across Eurasia (Rowlands et al., 2008; Njau et al., 2021). Genotype II ASFV was introduced into China in 2018, followed by the emergence of low-virulence genotype I strains and natural genotype I/II recombinants (Sun et al., 2021; Xin et al., 2023; Zhang et al., 2023; Zhao et al., 2023; Ding et al., 2024; Li and Zheng, 2025). These evolving strains pose growing challenges to ASF control and threaten global food security.

ASFV, the sole member of the genus *Asfivirus* and the only known DNA arbovirus, is a large icosahedral nucleocytoplasmic large DNA virus (NCLDV) (Iyer et al., 2001; Dixon et al., 2013; Wang et al., 2019; Yang et al., 2023; Yang et al., 2024). It possesses a complex five-layered architecture composed of an outer envelope, capsid, inner envelope, core shell, and nucleoid, as well as an ~187 kb genome encoding more than 160 proteins involved in viral replication, assembly, transport, and immune modulation (Salas and Andrés, 2013; Liu et al., 2019; Andrés et al., 2020; Yang et al., 2023). However, the complexity of its architecture and the multifunctionality of these proteins leave many mechanisms poorly understood, posing significant challenges for vaccine and antiviral development (Borca et al., 2020; Huang et al., 2025).

ASFV *B169L* encodes a small type III membrane protein that predominantly localizes to the endoplasmic reticulum (Gladue et al., 2024). Its highly conserved hairpin transmembrane domain exhibits viroporin activity, enabling the formation of ion-permeable channels that disrupt intracellular Ca^2+^ homeostasis. This activity promotes viral replication and modulates host responses, including the activation of inflammasome signaling via Ca^2+^ imbalance (Li et al., 2025). In addition, its early–late transcription pattern and high degree of conservation suggest that it plays a critical role throughout infection (Cackett et al., 2020; Cao et al., 2024). Overall, pB169L is a multifunctional viral factor that links membrane remodeling, ion regulation, and host immune modulation, making it a potential antiviral target and diagnostic biomarker.

In the present study, a monoclonal antibody against pB169L was generated through prokaryotic expression and purification of recombinant pB169L’, hybridoma screening, and systematic characterization via indirect immunofluorescence assays, Western blotting, and structural prediction. This antibody is intended to serve as a tool for dissecting the functions of pB169L in ASFV replication, viral morphogenesis, and immune evasion.

## 2 Materials and methods

### 2.1 Cells, viruses, and animals

WSL-R4 cells and primary porcine alveolar macrophages (PAMs) isolated from 1-month-old SPF piglets were maintained in RPMI-1640 medium supplemented with 10% fetal bovine serum (FBS; Gibco, USA). SP2/0 myeloma cells were cultured in Dulbecco’s modified Eagle’s medium (DMEM; Gibco, USA) supplemented with 20% FBS. The genotype II ASFV strain CADC_HN09 (ASFV-HN09; GenBank No. MZ614662.1) was used as the model virus in this study. All ASFV-related procedures were performed in strict accordance with the biosafety regulations of the Biosafety Level 3 Laboratory at China Agricultural University. BALB/c mice were obtained from SPF Biotechnology Co., Ltd. (Beijing).

### 2.2 Construction of recombinant plasmids containing the ASFV gene *B169L*

To express and purify the immunogen, the amino acid sequences corresponding to residues 1–22 and 84–164 of pB169L were fused in tandem (in duplicate) using a flexible linker and subsequently cloned and inserted into the pET-28a vector via the restriction sites BamHI and XhoI, respectively. The corresponding PCR primers are listed in Table 1.The resulting construct was verified by DNA sequencing and designated pET-28a pB169L’. To identify the minimal antigenic epitope recognized by the monoclonal antibodies, a series of truncated fragments of pB169L’ were amplified by PCR and cloned and inserted into the pEGFP-C1 vector. The corresponding PCR primers are listed in Table 2.

**Table 1:** Primers used for pB169L’ construction.

Name	Sequences (5′-3′)	Positions
1F	CAGCAAATGGGTCGCGGATCCATGAATGTAGATTTTATTGCGGG	1–22aa
1R	ATTATAAATGTCGTTGAACGGTTCGCACGTATAAATTTTT	
2F	ACGTGCGAACCGTTCAACGACATTTATAATAAATCAAACATG	84–164aa + GGGGS
2R	ATTGCTTCCTCCTCCTCCATTATTATATTGAGAAGGCATAATTAC	
1’F	AATGGAGGAGGAGGAAGCAATGTAGATTTTATTGCGGGTA	GGGGS+1–22aa
1’R	ATTATAAATGTCGTTGAACGGTTCGCACGTATAAATTTTT	
2’F	ACGTGCGAACCGTTCAACGACATTTATAATAAATCAAAC	84–164aa
2’R	GTGGTGGTGGTGGTGCTCGAGATTATTATATTGAGAAGGC	

**Table 2:** Primers for constructing truncated variants.

Name	Sequences (5′-3′)
GFP-B169L-F	AATTCTGCAGTCGACGGTACCGCCACCATGAATGTGGACTTCATCGCCG
GFP-B169L-R	TTATCTAGATCCGGTGGATCCTTAGTTGTTATACTGAGAAGGCATGATCAC
P1-F	AATTCTGCAGTCGACGGTACCATGAATGTGGACTTCATCGCCG
P1-R	TTATCTAGATCCGGTGGATCCTTAGTTGCTCTTGTTGTAGATGTCGTTGAAGGGCTCGCAGGTGTAGATC
P2-F	AATTCTGCAGTCGACGGTACCATGAAGATCTACACCTGCGAGCCCTTCAACGACATCTACAACAAGAGCAAC
P2-R	TTATCTAGATCCGGTGGATCCTTAGATGATCTCATCGCCTCCCTT
P3-F	AATTCTGCAGTCGACGGTACCATGCACGATAAGTACAAGGGAGGCG
P3-R	TTATCTAGATCCGGTGGATCCTTAGATCTTCTTGGGCTGATCCTCC
P4-F	AATTCTGCAGTCGACGGTACCATGAACGAGCTGGAGGAGGATCAG
P4-R	TTATCTAGATCCGGTGGATCCTTACAGGGGCTTGGAGTCGGG
P5-F	AATTCTGCAGTCGACGGTACCATGAAGCCCGTGAGCCTGCCC
P5-R	TTATCTAGATCCGGTGGATCCTCAGTTGTTATACTGAGAAGGCATGA
P5-1-F	GAACCGTCAGATCCGCTAGCGCTACCGGTCGCCACCATGG
P5-1-R	TTATCTAGATCCGGTGGATCCCACTTCCTGCAGAGGCACCAGGGGCTTGGAGTCGGGCAGGCTCACGGGCTTCGGGCCCGCGGTACCGTC
P5-2-F	GAACCGTCAGATCCGCTAGCGCTACCGGTCGCCACCATGG
P5-2-R	TTATCTAGATCCGGTGGATCCTCAGTTGTTATACTGAGAAGGCATGATCACTTCCTGCAGAGGCACCAGGGGCGGGCCCGCGGTACCGTC
P5-1-1-F	CCGCCCGTGAGCCTGCCCGACTCCAAGCCCCTGGT
P5-1-1-R	TCGGGCAGGCTCACGGGCGGGCCCGCGGTACCGTC
P5-1-2-F	CCCGGTGAGCCTGCCCGACTCCAAGCCCCTGGTGC
P5-1-2-R	AGTCGGGCAGGCTCACCGGGCCCGCGGTACCGTC
P5-1-3-F	CCGAGCCTGCCCGACTCCAAGCCCCTGGTGCCTCT
P5-1-3-R	TTGGAGTCGGGCAGGCTCGGGCCCGCGGTACCGTC
P5-1-4-F	CCCGCTGCCCGACTCCAAGCCCCTGGTGCCTCT
P5-1-4-R	TTGGAGTCGGGCAGCGGGCCCGCGGTACCGTCG
P5-1-5-F	CCGCCCGACTCCAAGCCCCTGGTGCCTCTGCAGGA
P5-1-5-R	AGGGGCTTGGAGTCGGGCGGGCCCGCGGTACCGTC
P5-1-6-F	CCGGACTCCAAGCCCCTGGTGCCTCTGCAGGAAGT
P5-1-6-R	ACCAGGGGCTTGGAGTCCGGGCCCGCGGTACCGTC
P5-1-7-F	CCGTCCAAGCCCCTGGTGCCTCTGCAGGAAGTGGGA
P5-1-7-R	AGGCACCAGGGGCTTGGACGGGCCCGCGGTACCGTC
P5-1-8-F	CCGAAGCCCCTGGTGCCTCTGCAGGAAGTGGGATC
P5-1-8-R	AGAGGCACCAGGGGCTTCGGGCCCGCGGTACCGTC
P5-1-9-F	CCGCCCCTGGTGCCTCTGCAGGAAGTGGGATCCAC
P5-1-9-R	TGCAGAGGCACCAGGGGCGGGCCCGCGGTACCGTC
P5-1-10-F	GGAAGGATCCACCGGATCTAGATAACTGATCATAA
P5-1-10-R	TAGATCCGGTGGATCCTTCCTGCAGAGGCACCAGG
P5-1-11-F	GCAGGGATCCACCGGATCTAGATAACTGATCATAA
P5-1-11-R	TAGATCCGGTGGATCCCTGCAGAGGCACCAGGGG
P5-1-12-F	TCTGGGATCCACCGGATCTAGATAACTGATCATAA
P5-1-12-R	TAGATCCGGTGGATCCCAGAGGCACCAGGGGCTT
P5-1-13-F	GCCTGGATCCACCGGATCTAGATAACTGATCATAA
P5-1-13-R	TAGATCCGGTGGATCCAGGCACCAGGGGCTTGGA
P5-1-14-F	GGTGGGATCCACCGGATCTAGATAACTGATCATAA
P5-1-14-R	TAGATCCGGTGGATCCCACCAGGGGCTTGGAGTCG
P5-1-15-F	CCTGGGATCCACCGGATCTAGATAACTGATCATAA
P5-1-15-R	TAGATCCGGTGGATCCCAGGGGCTTGGAGTCGGG
P5-1-16-F	GCCCGGATCCACCGGATCTAGATAACTGATCATAATC
P5-1-16-R	TCTAGATCCGGTGGATCCGGGCTTGGAGTCGGGCAG
P5-1-17-F	CAAGGGATCCACCGGATCTAGATAACTGATCATA
P5-1-17-R	AGATCCGGTGGATCCCTTGGAGTCGGGCAGGCT
P5-1-18-F	CTCCGGATCCACCGGATCTAGATAACTGATCATAA
P5-1-18-R	TCTAGATCCGGTGGATCCGGAGTCGGGCAGGCTCAC

### 2.3 Expression and purification of ASFV pB169L’ in prokaryotic cells

To obtain recombinant pB169L’, the plasmid pET-28a-pB169L’ was transformed into *E. coli* Rosetta (DE3) cells. The expression of the His-tagged recombinant protein was induced with 0.1 mM isopropyl β-D-1-thiogalactopyranoside (IPTG) for 8 h at 37 °C. SDS–PAGE analysis revealed that pB169L’ was predominantly expressed in the soluble fraction. After cell lysis by sonication and centrifugation at 8,000 rpm for 30 min, the supernatant was collected, filtered through a 0.22 μm membrane, and purified using Ni–NTA agarose affinity chromatography. The purified protein was subsequently dialyzed against phosphate-buffered solution. Protein expression and purity were confirmed by SDS–PAGE and Western blot analysis.

### 2.4 Preparation of monoclonal antibodies against ASFV pB169L

After emulsification of 30 μg of purified pB169L’ protein with Freund’s adjuvant, four 6–8-week-old female BALB/c mice were subcutaneously immunized, followed by three booster immunizations at 2-week intervals. Serum antibody titers were determined by ELISA and IFA 10 days after the third booster immunization. The mouse with the highest antibody titer was selected for the final boost immunization. Three days later, the splenocytes were collected and fused with SP2/0 cells at a ratio of 5:1 (splenocytes: SP2/0 cells) using 50% (w/v) PEG1450. Hybridomas were selected in semisolid HAT medium for 12 days. Positive clones were screened by ELISA, and antibody isotypes were determined using hybridoma culture supernatants and a mouse monoclonal antibody subclass ELISA kit. The selected 1B9 hybridoma cells were then used to produce ascites in BALB/c mice. Ascitic fluid was collected seven days after intraperitoneal inoculation after centrifugation to remove cellular debris, the ascitic fluid was filtered through a 0.22 μm filter membrane to remove impurities. Subsequently, IgG was purified by Protein A affinity chromatography.

### 2.5 Indirect immunofluorescence assay (IFA)

WSL-R4 cells grown on coverslips were subjected to plasmid transfection or viral infection. For transfection, cells at 80–90% confluence were transfected with the pEGFP-B169L plasmid. For infection, confluent cells were infected with ASFV at an MOI of 0.1. At the indicated times, the cells were fixed with cold 4% paraformaldehyde for 10 min, washed with PBS, permeabilized with 0.1% Triton X-100 for 10 min, and blocked with 1% BSA for 30 min. The samples were then incubated with primary antibodies (mouse anti-ASFV pB169L sera1-4 (1:100), rabbit anti-GFP (1:2000), rabbit anti-ASFV p54 (1:500) or p30 (1:2000), and mouse anti-ASFV pB169L 1B9 IgG (1:2000)) overnight at 4 °C. After incubation and washing, fluorescent secondary antibodies (Alexa Fluor™ 488 Goat anti-Rabbit or Goat anti-Mouse IgG (H + L) (1:1500), and Alexa Fluor™ 568 Goat anti-Rabbit or Goat anti-Mouse IgG (H + L) (1:1500)) were incubated at room temperature for 1 h. The nuclei were stained with DAPI, and the cells were imaged under a fluorescence microscope. After IFA staining and mounting with antifade medium, the samples were imaged using a Nikon A1 confocal microscope and analyzed with NIS-Elements software.

### 2.6 Western blot

WSL-R4 cells were transfected with pEGFP-B169L or infected with ASFV, and total cellular proteins were extracted at the indicated time points using RIPA lysis buffer. Protein samples were mixed with loading buffer, boiled for 10 min, separated by SDS–PAGE at an initial voltage of 80 V followed by 120 V, and then transferred onto PVDF membranes by wet transfer at 100 V for 120 min. Membranes were blocked with 5% skim milk for 2 h and washed with PBST. The membranes were then incubated with primary antibodies (mouse anti-His (1:2000), rabbit anti-GFP (1:2000), mouse anti-ASFV pB169L sera1-4 (1:100), rabbit anti-ASFV pp62 (1:1000), mouse anti-ASFV pB169L 1B9 IgG (1:2000), rabbit anti-ASFV p30 (1:2000), and mouse anti-β-actin (1:2000)) overnight at 4 °C, followed by incubation with HRP-conjugated secondary antibodies (1:5000). Protein signals were visualized using enhanced chemiluminescence (ECL) reagent and captured with a chemiluminescence imaging system.

### 2.7 Sequencing and bioinformatic analysis of antibody variable regions

To determine the molecular characteristics of 1B9 monoclonal antibody, the variable regions of its heavy and light chains were sequenced and analyzed. Briefly, total RNA was extracted from 1B9 hybridoma cells using TRIzol reagent according to the manufacturer’s instructions and then reverse-transcribed into cDNA. The cDNA was used as a template to amplify the immunoglobulin heavy-chain and light-chain variable region sequences. The amplified products were subjected to high-throughput sequencing. The sequencing data were further analyzed for sequence abundance and productivity, and the obtained variable region sequences were aligned against the IMGT database to determine V(D)J gene usage and annotate the framework regions (FRs) and complementarity-determining regions (CDRs). The highest-abundance productive sequences with complete variable region annotation were selected as the representative VH and VL sequences of 1B9 mAb.

### 2.8 Bioinformatic analysis of the pB169L protein

MEGA v7.0.26 was used to analyze the conservation of amino acid residues within the identified epitope by aligning the pB169L epitope sequence from the ASFV CADC_HN09 strain with the corresponding regions from 20 distinct ASFV strains. The secondary structure of pB169L was analyzed using DNASTAR v7.1. The three-dimensional structure of pB169L was predicted using I-TASSER to assess its spatial conformation. The identified epitope was then mapped onto the pB169L structural model using PyMOL v2.4.1, and its spatial features were further analyzed.

## 3 Results

### 3.1 Design, expression, and purification of a soluble pB169L immunogen

The TMHMM and IEDB online tools were used to predict the transmembrane domains and B-cell epitopes of pB169L. The results revealed that pB169L contains two high-confidence transmembrane domains, with extracellular regions located at both termini of the protein (Figure 1A). Notably, most predicted B-cell epitopes were distributed within these extracellular regions (Figure 1B). According to this analysis, amino acid residues 1–22 and 84–164 were selected to construct the plasmid pET-28a-pB169L’ for immunogen expression.

**Figure 1:**
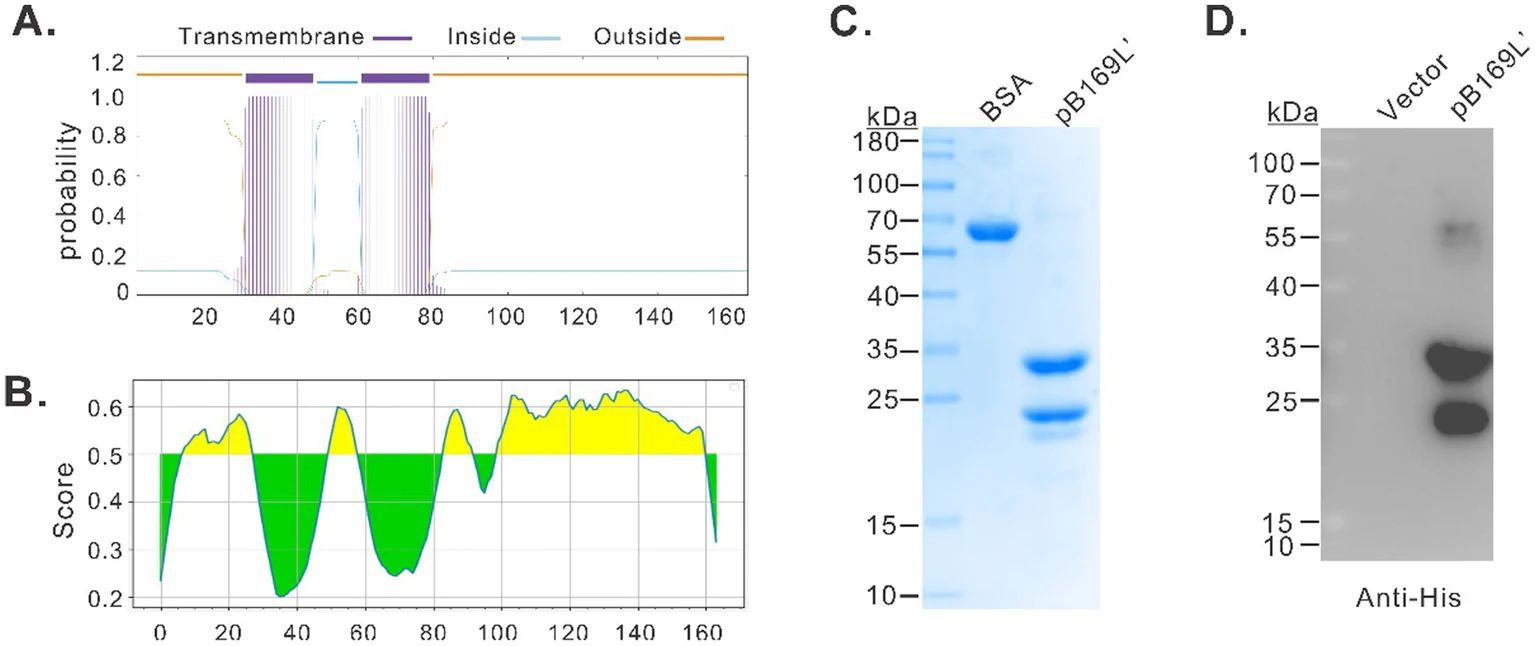
Expression and purification of ASFV pB169L’. **(A)** Transmembrane domain prediction of pB169L using TMHMM. **(B)** Prediction of B-cell epitopes in pB169L using IEDB. **(C)** Identification of the purified recombinant pB169L’ by SDS–PAGE. **(D)** Identification of recombinant pB169L’ by Western blot (WB) using an anti-His monoclonal antibody.

SDS–PAGE analysis revealed that recombinant pB169L’ was present mainly in the soluble fraction (Supplementary Figure S1). The protein was subsequently purified from the supernatant using Ni-NTA beads. SDS–PAGE analysis revealed a prominent band at approximately 25–35 kDa, which was consistent with the expected molecular weight, and Western blot (WB) confirmed specific reactivity with the anti-His antibody (Figures 1C,D). These results demonstrate that pB169L’ was successfully expressed and purified, providing a suitable immunogen for subsequent immunization.

### 3.2 Immunogenicity determination of the recombinant pB169L’ protein

After four immunizations with pB169L’, serum samples were collected and analyzed by ELISA. All four mice developed high antibody titers (>1:25,600), with mouse No. 3 showing the highest titer (Figure 2A). To further assess serum reactivity, Western blot (WB) and immunofluorescence assay (IFA) were performed under both transfection and infection conditions. The results revealed that all four serum samples specifically recognized pB169L in WSL-R4 cells transiently expressing GFP-B169L (Figures 2B,C). Consistently, endogenous pB169L could also be detected by the immune sera in ASFV-infected WSL-R4 cells (Figures 2D–G). These results demonstrate that immunization with pB169L’ successfully induced specific antibodies against pB169L. Furthermore, WB analysis revealed that these serum samples could recognize the predicted product of pB169L, which was approximately 18 kDa in size. Unexpectedly, they also reacted with a slow-moving species that had a molecular weight of approximately 21 kDa (Figure 2D). Notably, the IFA results revealed that pB169L colocalized with the viral inner envelope protein p54, a marker of viral factories, rather than the viral early protein p30 (Figures 2F,G), suggesting that pB169L was predominantly localized in viral factories.

**Figure 2:**
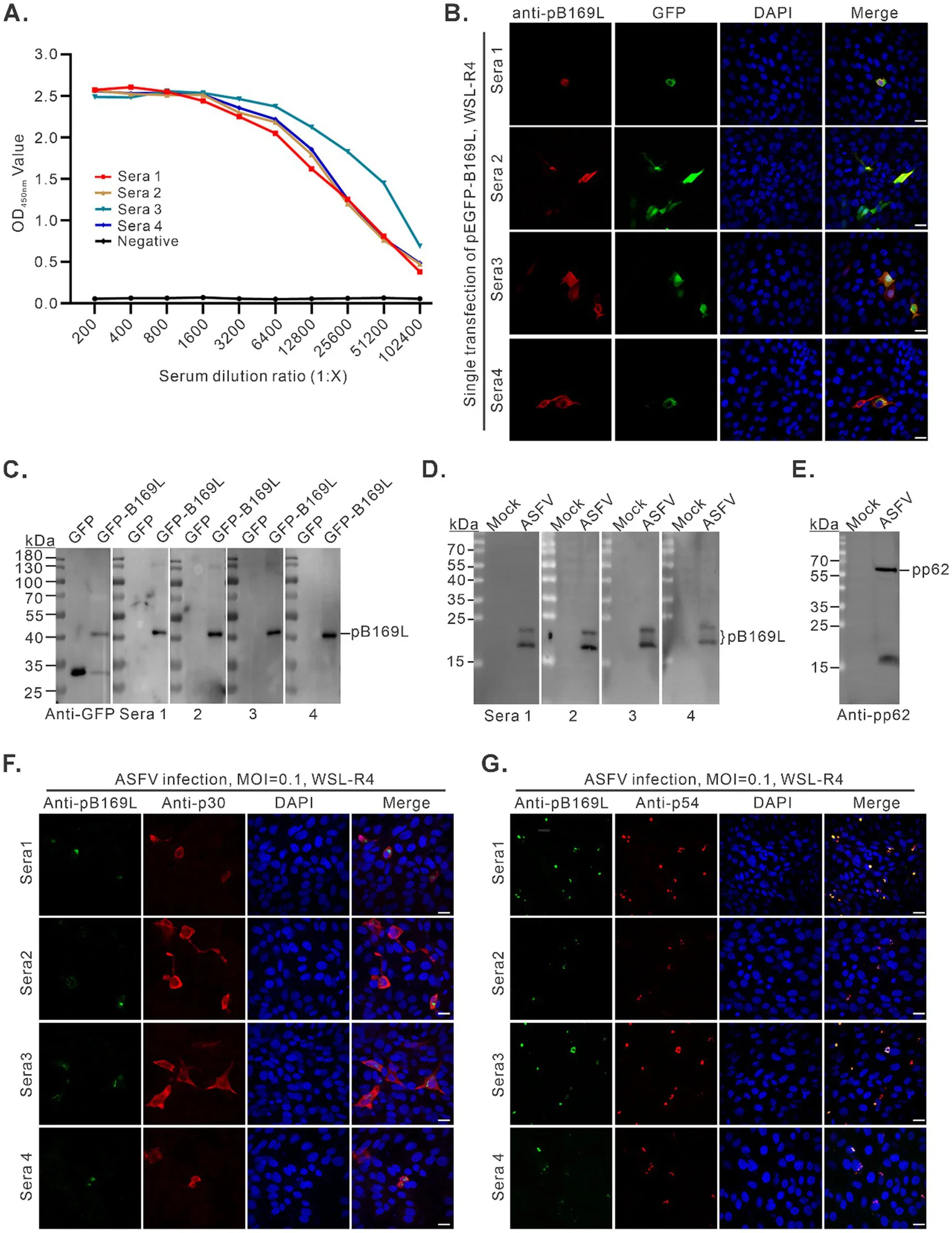
Characterization of sera from mice immunized with pB169L’. **(A)** Antibody titers in mouse serum samples were determined by ELISA. Blood samples were collected from the orbital sinuses of the mice on day 10 following the third booster immunization. ELISA plates were coated with 2 μg/mL pB169L’ protein, the test sera were serially diluted in a 2-fold gradient starting from a 200-fold dilution, and a 200-fold dilution of negative serum was used as the negative control. **(B,C)** The reactivity of the sera with GFP-B169L was analyzed by IFA and WB under transfection conditions. GFP-B169L was transiently expressed in WSL-R4 cells, and the samples were harvested at 24 h post-transfection and subjected to IFA or WB using anti-GFP antibodies and mouse sera. **(D,E)** The reactivity of the sera with endogenous pB169L was analyzed by WB under infection conditions. WSL-R4 cells were infected with ASFV HN09 at an MOI of 0.1. At 24 h post-infection (hpi), the cells were harvested for WB using sera and antibodies against the viral protein pp62. The ASFV structural protein pp62 served as a marker of infection. **(F,G)** Same as in D and E, except that cells were fixed for IFA using sera and antibodies against viral p30 or p54. The ASFV structural proteins p30 and p54 were used as markers of infection and viral factories, respectively. Scale bar, 20 μm (applies to all panels unless otherwise indicated).

### 3.3 Functional characterization and isotype identification of pB169L monoclonal antibody 1B9

Based on the above results, mouse No. 3 was selected for the final booster immunization. Splenocytes were fused with SP2/0 cells and screened in HAT-containing medium. After three rounds of subcloning and ELISA screening, one positive hybridoma clone was obtained and named 1B9. Isotype analysis revealed that 1B9 belonged to the IgG1 heavy-chain subclass with a kappa light chain (Figure 3A). The specificity of 1B9 was confirmed by WB and IFA under transfection conditions. In WSL-R4 cells transfected with pEGFP-B169L, both the 1B9 antibody and the anti-GFP antibody detected GFP-B169L by WB (Figure 3B). Consistently, the IFA results revealed that the signal detected by 1B9 showed strong overlap with GFP fluorescence in the cytoplasm (Figure 3C), confirming the specificity of the monoclonal antibody 1B9.

**Figure 3:**
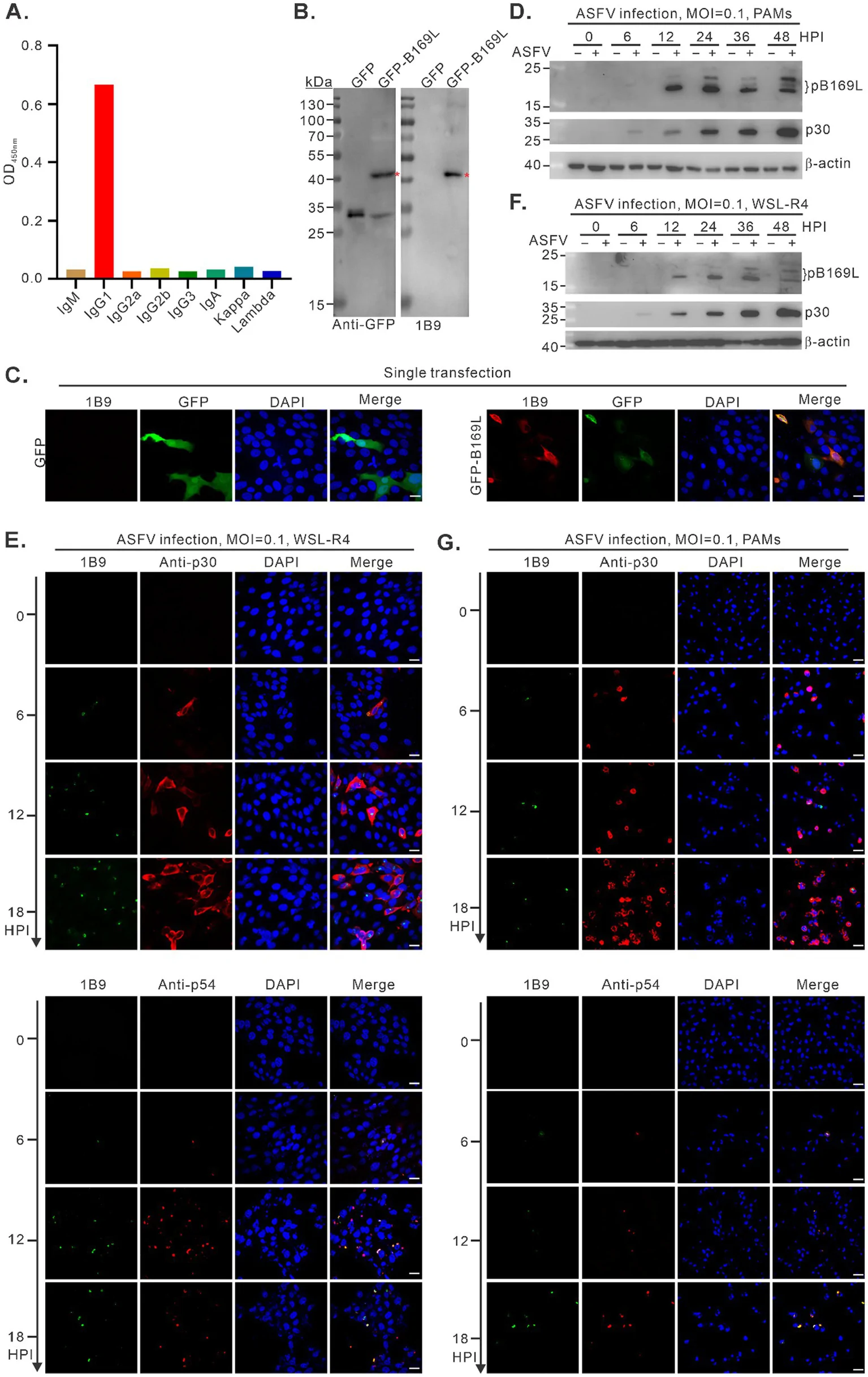
Identification of pB169L mAb 1B9. **(A)** Isotype determination of the anti-pB169L monoclonal antibody 1B9 by capture ELISA. ELISA plates were coated with goat anti-mouse immunoglobulin antibody, followed by incubation with hybridoma culture supernatant and subsequent detection with isotype-specific anti-mouse immunoglobulin antibodies. **(B,C)** The reactivity of 1B9 with GFP-B169L was analyzed by IFA and WB under transfection conditions. GFP-B169L was transiently expressed in WSL-R4 cells, and the samples were harvested at 24 h post-transfection and subjected to IFA or WB using anti-GFP antibodies and 1B9. **(D,F)** The expression kinetics of pB169L in PAMs **(D)** and WSL-R4 cells **(F)** were analyzed by WB. WSL-R4 cells or PAMs were infected with ASFV HN09 at an MOI of 0.1. At the indicated time points, the cells were harvested for WB using 1B9 and antibodies against the viralprotein p30. The ASFV protein p30 served as a marker of infection. **(E,G)** Dynamic changes in the subcellular localization of pB169L were determined by IFA. Same as in D and F, except that cells were fixed for IFA using 1B9 and antibodies to viral p30 or p54. The ASFV structural proteins p30 and p54 were used as markers of infection and viral factories, respectively. Scale bar, 20 μm (applies to all panels unless otherwise indicated).

Next, the dynamic changes in pB169L expression and subcellular localization were analyzed by WB and IFA in ASFV-infected PAMs and WSL-R4 cells with genotype II ASFV HN09 strain at an MOI of 0.1. Two specific bands were also detected by the monoclonal antibody in infected cells at 12 hpi (Figures 3D,F). The 18-kDa pB169L is the predominant form, while the abundance of the 21-kDa pB169L is relatively low. By 48 hpi, the levels of the two pB169L forms were comparable in both cell types (Figures 3D,F). IFA further revealed that pB169L was detectable in cytoplasmic viral factories at 6 hpi and markedly increased at 18 hpi in both ASFV-infected WSL-R4 cells and PAMs (Figures 3E,G). Overall, the expression patterns of pB169L were similar in the two cell types; pB169L colocalized with p54 in viral factories and was expressed during the mid-to-late stage of ASFV infection.

### 3.4 Molecular definition of the 1B9 variable regions

To further define the molecular identity of 1B9 mAb, the variable regions of its heavy and light chains were sequenced and subjected to IMGT analysis. Productive VH and κ-type VL sequences were identified. The VH sequence was assigned to IGHV7-3*02, IGHJ2*01, and IGHG1*01, with IGHD5-2*01, IGHD5-3*01, or IGHD5-4*01 as candidate D genes, whereas the VL sequence was assigned to IGKV8-30*01, IGKJ1*01, and IGKC*02. These results confirmed the molecular features of 1B9 mAb and provided defined VH/VL sequence information for further recombinant antibody construction or antibody engineering. The CDR amino acid sequences are listed in Table 3. The complete nucleotide and deduced amino acid sequences of the 1B9 VH and VL regions are provided in Supplementary Table S1.

**Table 3:** V(D)J gene usage and CDR amino acid sequences of 1B9.

Chain	V gene	D gene	J gene	C gene	CDR1	CDR2	CDR3
VH	IGHV7-3*02	IGHD5-2*01 /IGHD5-3*01 /IGHD5-4*01	IGHJ2*01	IGHG1*01	GFTFTDYY	IRSKANGSTT	ARDSGGILDY
VL	IGKV8-30*01	—	IGKJ1*01	IGKC*02	QSLLYNGNKKNY	WAS	QQYYRYPWT

“—” indicates not applicable because immunoglobulin light chains are generated by V-J recombination without D gene usage.

### 3.5 Identifying the antigenic epitope recognized by the pB169L mAb

To determine the epitope of the monoclonal antibody, pB169L’ was segmented into five overlapping truncated fragments, as shown in Figure 4A: P1 (1–30 aa), P2 (15–49 aa), P3 (39–69 aa), P4 (59–89 aa), and P5 (79–103 aa). The constructs were separately cloned and inserted into the pEGFP-C1 vector and transfected into WSL-R4 cells for expression, with GFP and GFP-B169L acting as negative and positive controls, respectively. WB and IFA results demonstrated that 1B9 identified the complete pB169L protein and P5 but failed to recognize the P1, P2, P3, and P4 fragments (Figures 4B,C), indicating that the antigenic epitope is located between amino acids 79 and 103. Consequently, P5 was subsequently divided into two overlapping segments, P5-1 (79–95 aa) and P5-2 (87–103 aa) (Figure 4A). We found that 1B9 could react with P5-1 but not P5-2 (Figures 4D,E).

**Figure 4:**
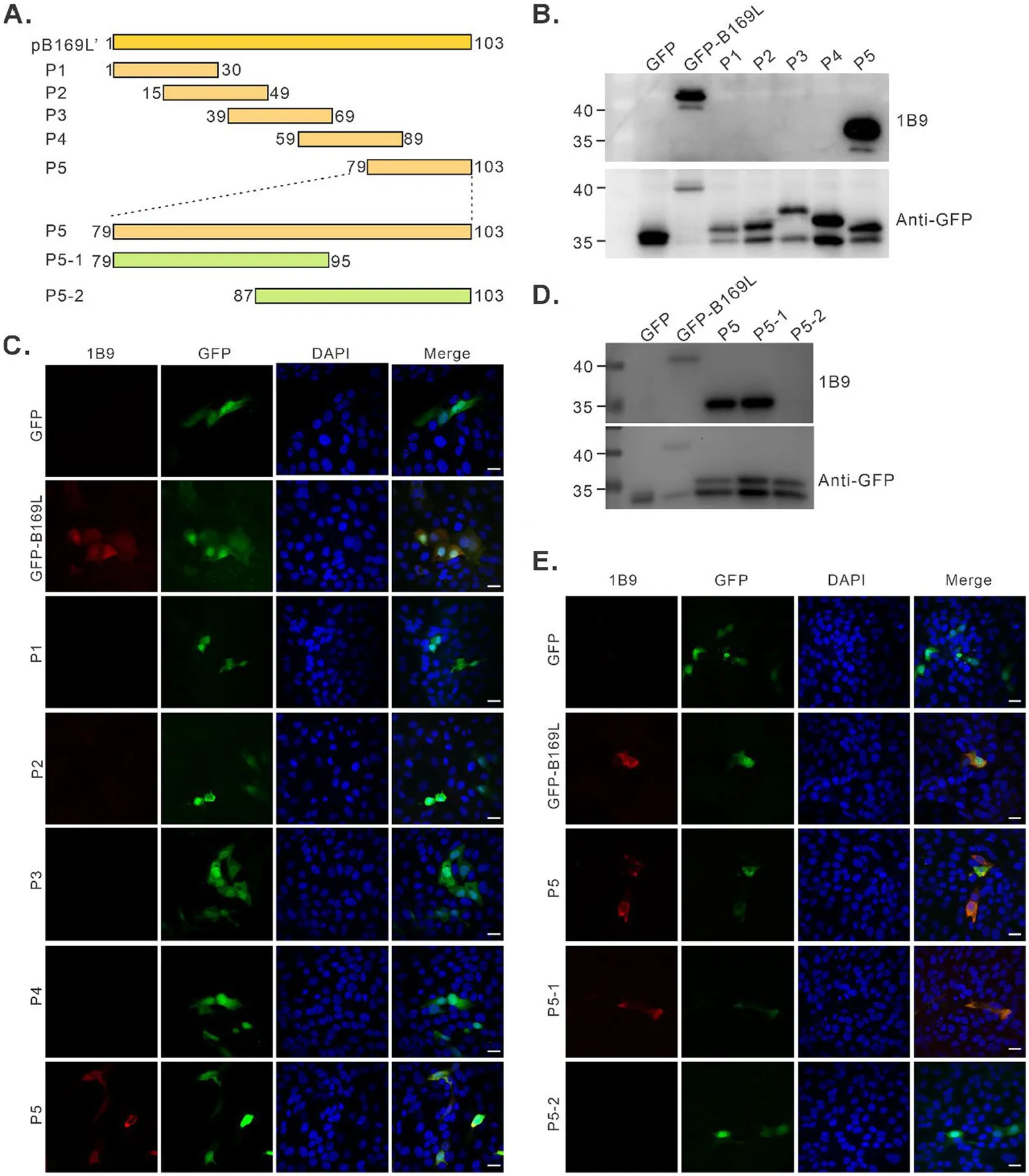
Identification of antigenic epitope in pB169L. **(A)** Schematic diagram of the two rounds of truncation used for epitope mapping of pB169L’. Orange and green indicate the first and second rounds, respectively. **(B,C)** First-round truncation variants of pB169L’ were analyzed by WB **(B)** and IFA **(C)**. Different truncated forms of pB169L’ were transiently expressed in WSL-R4 cells. At 24 h post-transfection, the cells were subjected to WB and IFA with the indicated antibodies. **(D,E)** Based on the results of the first round, the second-round truncated variants were further analyzed using the same methods as in **(B,C)**.

To further characterize the antigenic epitope, truncated variants were constructed by sequentially removing one amino acid from the N- and C-termini of P5-1. P5-1-1 through P5-1-9 are consecutive N-terminal truncation mutants, and P5-1-10 to P5-1-18 are consecutive C-terminal truncation mutants (Figure 5A). P5-1-7 (aa 86–95) retained reactivity, whereas P5-1-8 (aa 87–95) lost reactivity, confirming that the 86th residue marks the N-terminal boundary of the epitope. Conversely, P5-1-10 (aa 79–94) retained reactivity, whereas P5-1-11 (aa 79–93) lost reactivity, establishing the 94th residue as the C-terminal boundary (Figures 5B,C). Therefore, the epitope recognized by 1B9 comprises amino acids 86–94 of pB169L’, matching the sequence ^147^SKPLVPLQE^155^ in pB169L.

**Figure 5:**
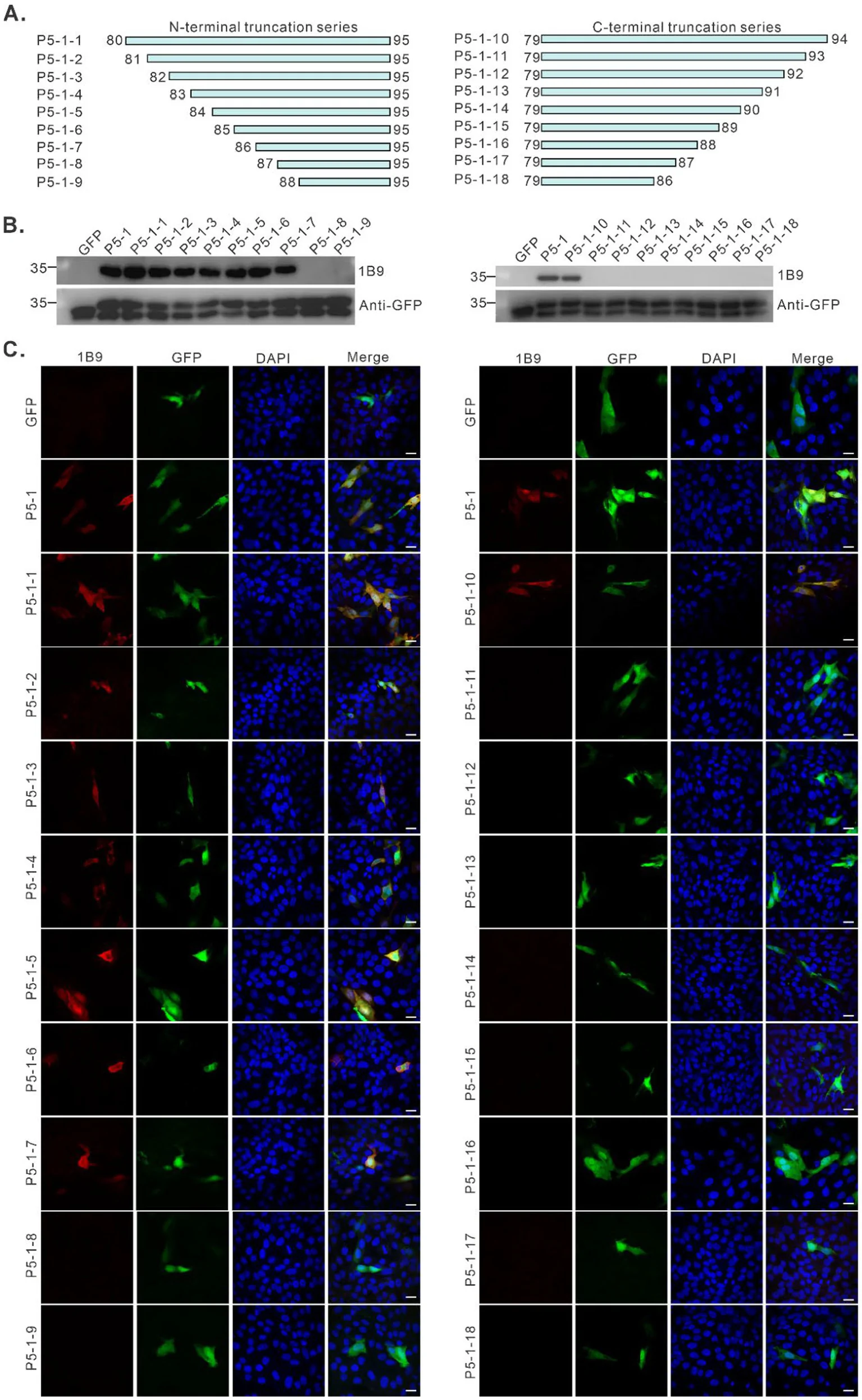
Further characterization of the pB169L antigenic epitope. **(A)** Schematic representation of the sequential truncation strategy used to define the minimal epitope within P5-1 (aa 79–95). P5-1-1 to P5-1-9 represent progressive N-terminal deletions, whereas P5-1-10 to P5-1-18 represent progressive C-terminal deletions. **(B,C)** As in Figures 4B,C, the truncated variants were analyzed by WB **(B)** and IFA **(C)** using anti-GFP and anti-pB169L antibodies.

### 3.6 Bioinformatics analysis of the pB169L antigenic epitope

To evaluate the conservation of the pB169L antigenic epitope among ASFV genotypes, sequence alignment was performed between the reference strain (CADC_HN09) and 20 additional isolates representing 11 genotypes retrieved from GenBank. The epitope was highly conserved among genotypes I, II, III, VII, and VIII. In contrast, single amino acid substitutions were observed at position 147 in genotype V, position 150 in genotype XV, and position 151 in genotypes IX and X. Notably, the pB169L sequence of the reference strain shared less than 10% similarity with genotypes IV and XX (Figure 6A).

**Figure 6:**
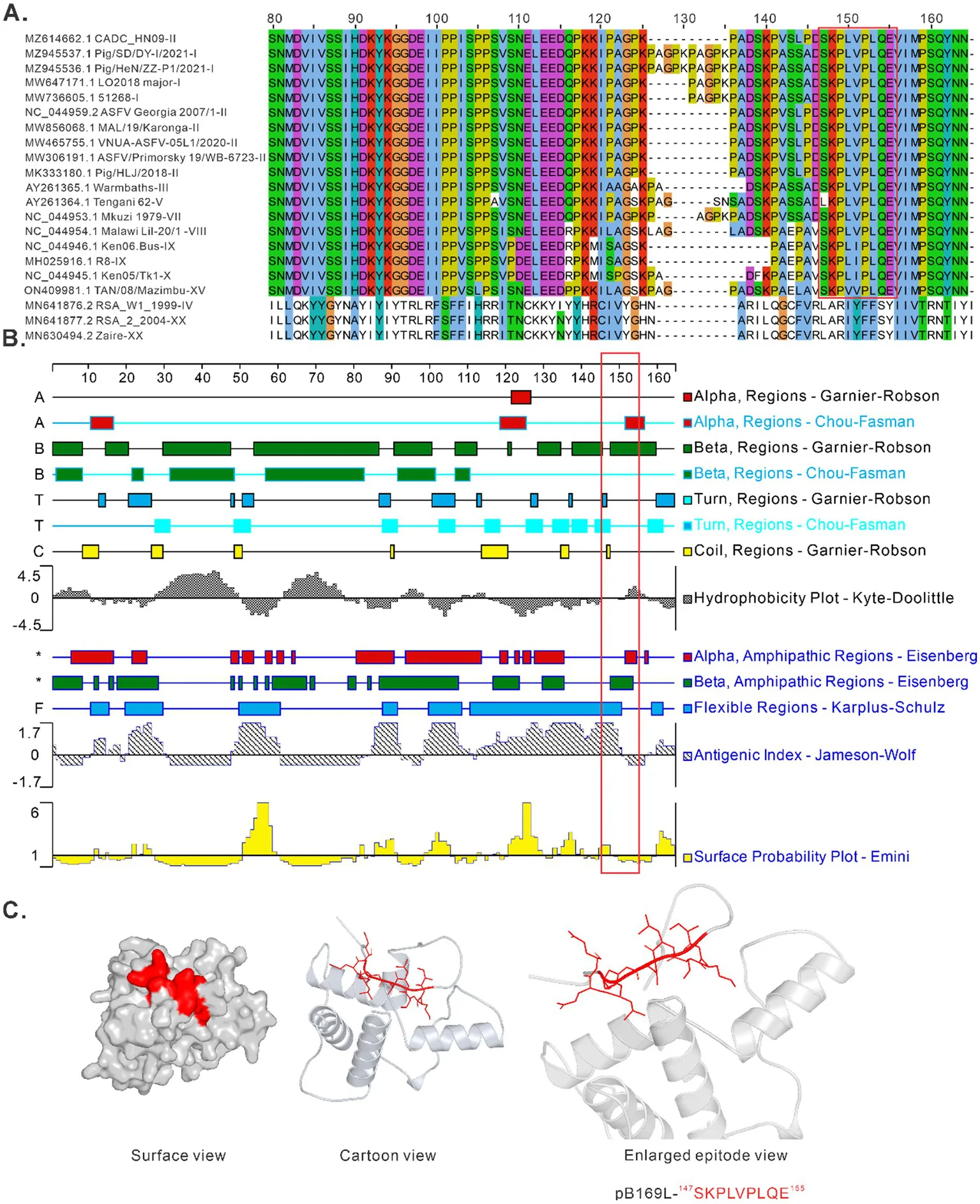
Bioinformatics analysis of the pB169L antigenic epitope. **(A)** Conservation analysis of the pB169L epitope. Sequence alignment of pB169L (UFD97845.1) with representative ASFV strains. **(B)** Secondary structure prediction of pB169L using DNASTAR v7.1 software. **(C)** Localization of the antigenic epitope. I-TASSER (https://zhanggroup.org/I-TASSER/), an online protein structure prediction tool, was used to estimate the three-dimensional structure of the pB169L protein. PyMOL software was used to map the discovered epitope in a simulated three-dimensional model of the pB169L protein.

Secondary-structure and antigenicity analyses indicated that the epitope region ^147^SKPLVPLQE^155^ is hydrophilic, flexible, and antigenic, supporting its potential as an antigenic epitope (Figure 6B). Moreover, the three-dimensional structure of pB169L was predicted using I-TASSER software and visualized with PyMOL. The results revealed that the epitope ^147^SKPLVPLQE^155^ was located on the protein surface and formed a curved/loop-like conformation, which is consistent with its accessibility to antibody binding (Figure 6C).

## 4 Discussion

African swine fever virus (ASFV) encodes numerous proteins with poorly defined functions, limiting the mechanistic understanding of viral replication and host interactions (Karger et al., 2019; Wang et al., 2021; Li et al., 2022; Dolata et al., 2023; Shen et al., 2024; Zhang et al., 2026). pB169L has been implicated in viral replication and immune regulation, suggesting a role in ASFV pathogenesis (Li et al., 2025). Recent studies have indicated that pB169L may contribute to viral replication and inflammatory responses, potentially through membrane-associated activities and the modulation of host signaling pathways (Gladue et al., 2024; Li et al., 2025). In this study, we generated a monoclonal antibody (mAb) against pB169L, which specifically recognizes the protein in both transfected and infected cells, providing a reliable tool for studying its expression and localization. Furthermore, the molecular characterization of the VH and VL regions of 1B9 has laid the foundation for future research on the preparation of recombinant antibodies and antibody engineering.

In this context, the identification of a conserved linear B-cell antigenic epitope (^147^SKPLVPLQE^155^) within pB169L is particularly noteworthy. Our bioinformatic and structural analyses demonstrated that this epitope is conserved among several epidemiologically important ASFV genotypes and is located on the protein surface and has favorable physicochemical properties, including hydrophilicity, flexibility, and high antigenicity. These features are consistent with those of classical B-cell epitopes and may facilitate antibody recognition. However, the observed variability in certain genotypes suggests potential genotype-specific antigenic differences, which may have implications for cross-reactivity and diagnostic or vaccine design.

Using 1B9, we detected pB169L from approximately 6 h post-infection by IFA and observed progressive accumulation in viral factories, where it colocalized with p54. p54 is a membrane-associated structural protein that is recruited to ASFV assembly sites and participates in the formation of viral membrane precursors (Rodriguez et al., 1994; Rodríguez et al., 2004). The spatial relationship between pB169L and p54 therefore supports a role for pB169L in membrane-associated stages of infection, although colocalization alone does not establish a direct interaction (Alejo et al., 2018; Li et al., 2025). The antibody should facilitate future proximity, co-immunoprecipitation, and immunoelectron-microscopy studies addressing this question.

Two electrophoretic pB169L species of approximately 18 and 21 kDa were detected in both PAMs and WSL-R4 cells. The 18-kDa band is close to the predicted molecular mass of full-length pB169L, whereas the origin of the slower-migrating species remains unresolved. The *B169L* locus is transcriptionally complex, and an alternative transcript associated with the adjacent *B438L* promoter has been described (Cao et al., 2024). However, whether this transcriptional complexity contributes to the two electrophoretic species observed here is unknown. Definitive characterization will require targeted biochemical and transcript-level analyses, including phosphatase/glycosidase treatment, mass spectrometry, mutagenesis, and/or transcript-specific expression analysis.

The present study is primarily a reagent-development and epitope-mapping investigation. It does not establish diagnostic sensitivity, protective activity, or neutralizing capacity, and these applications should not be inferred from sequence conservation alone. Rather, the major value of 1B9 lies in enabling reproducible molecular studies of pB169L abundance, localization, structural forms, and protein interactions during ASFV infection.

## 5 Conclusion

A mouse monoclonal antibody, 1B9, was developed against the ASFV viroporin pB169L and validated for immunoblotting and immunofluorescence. The antibody variable regions were molecularly defined, and the recognized linear epitope was mapped to residues 147–155 (SKPLVPLQE) in the C-terminal region of pB169L. The 1B9 antibody enabled detection of the progressive accumulation of pB169L in viral factories during infection and identified two electrophoretic pB169L species in infected cells. This antibody and its sequence information provide practical tools for future biochemical, structural, and cell-biological investigation of pB169L.

## Data Availability

The datasets presented in this study can be found in online repositories. The names of the repository/repositories and accession number(s) can be found in the article/Supplementary material.

## References

[ref1] Alejo A. Matamoros T. Guerra M. Andrés G. (2018). A proteomic atlas of the African swine fever virus particle. J. Virol. 92:e01293–18. doi: 10.1128/jvi.01293-18, 30185597 PMC6232493

[ref2] Andrés G. Charro D. Matamoros T. Dillard R. S. Abrescia N. G. A. (2020). The cryo-EM structure of African swine fever virus unravels a unique architecture comprising two icosahedral protein capsids and two lipoprotein membranes. J. Biol. Chem. 295, 1–12. doi: 10.1074/jbc.AC119.011196, 31649031 PMC6952596

[ref3] Borca M. V. Ramirez-Medina E. Silva E. Vuono E. Rai A. Pruitt S. . (2020). Development of a highly effective African swine fever virus vaccine by deletion of the I177L gene results in sterile immunity against the current epidemic Eurasia strain. J. Virol. 94:e02017–19. doi: 10.1128/jvi.02017-19, 31969432 PMC7081903

[ref4] Cackett G. Matelska D. Sýkora M. Portugal R. Malecki M. Bähler J. . (2020). The African swine fever virus transcriptome. J. Virol. 94:e00119–e00120. doi: 10.1128/jvi.00119-20, 32075923 PMC7163114

[ref5] Cao H. W. Deng H. Wang Y. J. Liu D. Q. Li L. F. Li M. L. . (2024). The distal promoter of the B438L gene of African swine fever virus is responsible for the transcription of the alternatively spliced B169L. Viruses 16:1058. doi: 10.3390/v16071058, 39066221 PMC11281499

[ref6] Ding L. L. Wang Z. G. Wang T. P. Zhang Z. J. Jiang F. W. Song L. F. . (2024). African swine fever virus in China: the diagnostic target gene (B646L) has acquired multi-point mutations. J. Infect. 89:106351. doi: 10.1016/j.jinf.2024.106351, 39551245

[ref7] Dixon L. K. Chapman D. A. G. Netherton C. L. Upton C. (2013). African swine fever virus replication and genomics. Virus Res. 173, 3–14. doi: 10.1016/j.virusres.2012.10.020, 23142553

[ref8] Dolata K. M. Pei G. Netherton C. L. Karger A. (2023). Functional landscape of African swine fever virus-host and virus-virus protein interactions. Viruses 15:1634. doi: 10.3390/v15081634, 37631977 PMC10459248

[ref9] Fuchs W. Assad-Garcia N. Abkallo H. M. Xue Y. Oldfield L. M. Fedorova N. . (2025). A synthetic genomics-based African swine fever virus engineering platform. Sci. Adv. 11:eadu7670. doi: 10.1126/sciadv.adu7670, 40138431 PMC11939070

[ref10] Gladue D. P. Gomez-Lucas L. Largo E. Ramirez-Medina E. Torralba J. Queralt-Martín M. . (2024). Viroporin-like activity of the hairpin transmembrane domain of African swine fever virus B169L protein. J. Virol. 98:e00231–e00224. doi: 10.1128/jvi.00231-24, 38980063 PMC11334534

[ref11] Huang R. J. Luo R. Lan J. Lu Z. H. Qiu H. J. Wang T. . (2025). The multigene family genes-encoded proteins of African swine fever virus: roles in evolution, cell tropism, immune evasion, and pathogenesis. Viruses 17:865. doi: 10.3390/v17060865, 40573456 PMC12197721

[ref12] Iyer L. M. Aravind L. Koonin E. V. (2001). Common origin of four diverse families of large eukaryotic DNA viruses. J. Virol. 75, 11720–11734. doi: 10.1128/jvi.75.23.11720-11734.2001, 11689653 PMC114758

[ref13] Karger A. Pérez-Núñez D. Urquiza J. Hinojar P. Alonso C. Freitas F. B. . (2019). An update on African swine fever virology. Viruses 11:864. doi: 10.3390/v11090864 PMC6784044 31533244

[ref14] Li Z. Y. Chen W. X. Qiu Z. L. Li Y. W. Fan J. D. Wu K. K. . (2022). African swine fever virus: a review. Life 12:1255. doi: 10.3390/life12081255 PMC9409812 36013434

[ref15] Li J. Q. Li Q. G. Wang Y. J. Guo Z. H. Qu Y. X. Wang X. . (2025). The B169L protein of African swine fever virus functions as a viroporin that activates the calcium-mediated inflammasome. PLoS Pathog. 21:e1013686. doi: 10.1371/journal.ppat.1013686, 41237200 PMC12638030

[ref16] Li S. Wang T. Luo Y. Li L. Wang Y. Song H. . (2026). Centennial African swine fever: a comprehensive overview. Animals Zoonoses 2, 111–124. doi: 10.1016/j.azn.2026.01.006

[ref17] Li M. Zheng H. X. (2025). Insights and progress on epidemic characteristics, pathogenesis, and preventive measures of African swine fever virus: a review. Virulence 16:2457949. doi: 10.1080/21505594.2025.2457949, 39937724 PMC11901552

[ref18] Liu S. Luo Y. Z. Wang Y. J. Li S. H. Zhao Z. N. Bi Y. H. . (2019). Cryo-EM structure of the African swine fever virus. Cell Host Microbe 26, 836–843.e3. doi: 10.1016/j.chom.2019.11.004, 31787524

[ref19] Montgomery R. E. (1921). On a form of swine fever occurring in British East Africa (Kenya Colony). J. Comp. Pathol. Ther. 34, 159–191. doi: 10.1016/S0368-1742(21)80031-4

[ref20] Njau E. P. Entfellner J. B. D. Machuka E. M. Bochere E. N. Cleaveland S. Shirima G. M. . (2021). The first genotype II African swine fever virus isolated in Africa provides insight into the current Eurasian pandemic. Sci. Rep. 11:13081. doi: 10.1038/s41598-021-92593-2 PMC8219699 34158551

[ref21] Rodriguez F. Alcaraz C. Eiras A. Yanez R. J. Rodriguez J. M. Alonso C. . (1994). Characterization and molecular basis of heterogeneity of the African swine fever virus envelope protein p54. J. Virol. 68, 7244–7252. doi: 10.1128/jvi.68.11.7244-7252.1994, 7933107 PMC237164

[ref22] Rodríguez J. M. García-Escudero R. Salas M. L. Andrés G. (2004). African swine fever virus structural protein p54 is essential for the recruitment of envelope precursors to assembly sites. J. Virol. 78, 4299–4313. doi: 10.1128/jvi.78.8.4299-4313.2004, 15047843 PMC374266

[ref23] Rowlands R. J. Michaud V. Heath L. Hutchings G. Oura C. Vosloo W. . (2008). African swine fever virus isolate, Georgia, 2007. Emerg. Infect. Dis. 14, 1870–1874. doi: 10.3201/eid1412.080591, 19046509 PMC2634662

[ref24] Salas M. L. Andrés G. (2013). African swine fever virus morphogenesis. Virus Res. 173, 29–41. doi: 10.1016/j.virusres.2012.09.016, 23059353

[ref25] Shen D. D. Zhang G. G. Weng X. G. Liu R. Q. Liu Z. H. Sheng X. P. . (2024). A genome-wide CRISPR/Cas9 knockout screen identifies TMEM239 as an important host factor in facilitating African swine fever virus entry into early endosomes. PLoS Pathog. 20:e1012256. doi: 10.1371/journal.ppat.1012256, 39024394 PMC11288436

[ref26] Sun E. C. Huang L. Y. Zhang X. F. Zhang J. W. Shen D. D. Zhang Z. J. . (2021). Genotype I African swine fever viruses emerged in domestic pigs in China and caused chronic infection. Emerg. Microbes Infect. 10, 2183–2193. doi: 10.1080/22221751.2021.1999779, 34709128 PMC8635679

[ref27] Wang Y. Kang W. F. Yang W. P. Zhang J. Li D. Zheng H. X. (2021). Structure of African swine fever virus and associated molecular mechanisms underlying infection and immunosuppression: a review. Front. Immunol. 12:715582. doi: 10.3389/fimmu.2021.715582, 34552586 PMC8450572

[ref28] Wang N. Zhao D. M. Wang J. L. Zhang Y. L. Wang M. Gao Y. . (2019). Architecture of African swine fever virus and implications for viral assembly. Science 366, 640–644. doi: 10.1126/science.aaz1439, 31624094

[ref29] Xin G. Y. Kuang Q. Y. Le S. J. Wu W. C. Gao Q. Gao H. . (2023). Origin, genomic diversity and evolution of African swine fever virus in East Asia. Virus Evol. 9:vead060. doi: 10.1093/ve/vead060 PMC10590196 37868933

[ref30] Yang S. C. Miao C. Liu W. Zhang G. L. Shao J. J. Chang H. Y. (2023). Structure and function of African swine fever virus proteins: current understanding. Front. Microbiol. 14:1043129. doi: 10.3389/fmicb.2023.1043129, 36846791 PMC9950752

[ref31] Yang J. Shao Z. W. Zhao X. Zhang W. Z. Zhang Y. X. Li L. X. . (2024). Structures of African swine fever virus topoisomerase complex and their implications. Nat. Commun. 15:6484. doi: 10.1038/s41467-024-50981-y PMC11294524 39090127

[ref32] Yang Y. Yuan H. X. Zhang Y. L. Luan J. Wang H. L. (2025). Progress in African swine fever vector vaccine development. Int. J. Mol. Sci. 26:921. doi: 10.3390/ijms26030921, 39940691 PMC11816837

[ref33] Zhang D. Y. Liu B. H. Liu H. N. Mao R. Q. Cao W. J. Zhang X. L. . (2026). Quantitative proteomic analysis identifies the unfolded protein response as a host pathway co-opted by ASFV to promote replication. mBio 17:e03242-25. doi: 10.1128/mbio.03242-25, 41347792 PMC12802178

[ref34] Zhang Y. Wang Q. H. Zhu Z. Y. Wang S. J. Tu S. Y. Zhang Y. Q. (2023). Tracing the origin of genotype II African swine fever virus in China by genomic epidemiology analysis. Transbound. Emerg. Dis.:4820809. doi:10.1155/2023/4820809 PMC12017148 40303812

[ref35] Zhao D. M. Sun E. C. Huang L. Y. Ding L. L. Zhu Y. M. Zhang J. W. . (2023). Highly lethal genotype I and II recombinant African swine fever viruses detected in pigs. Nat. Commun. 14:3096. doi: 10.1038/s41467-023-38868-w, 37248233 PMC10226439

