# Supplementary material for: Monoclonal antibody 1B9 against the African swine fever virus viroporin pB169L: molecular characterization and epitope mapping
Source: Front Microbiol. 2026 Sep 22;17:1942750. doi: 10.3389/fmicb.2026.1942750 (PMC13638613; doi:10.3389/fmicb.2026.1942750)
Supplement: Supplementary file 1 [file Supplementary_file_1.pdf]

Supplemental data

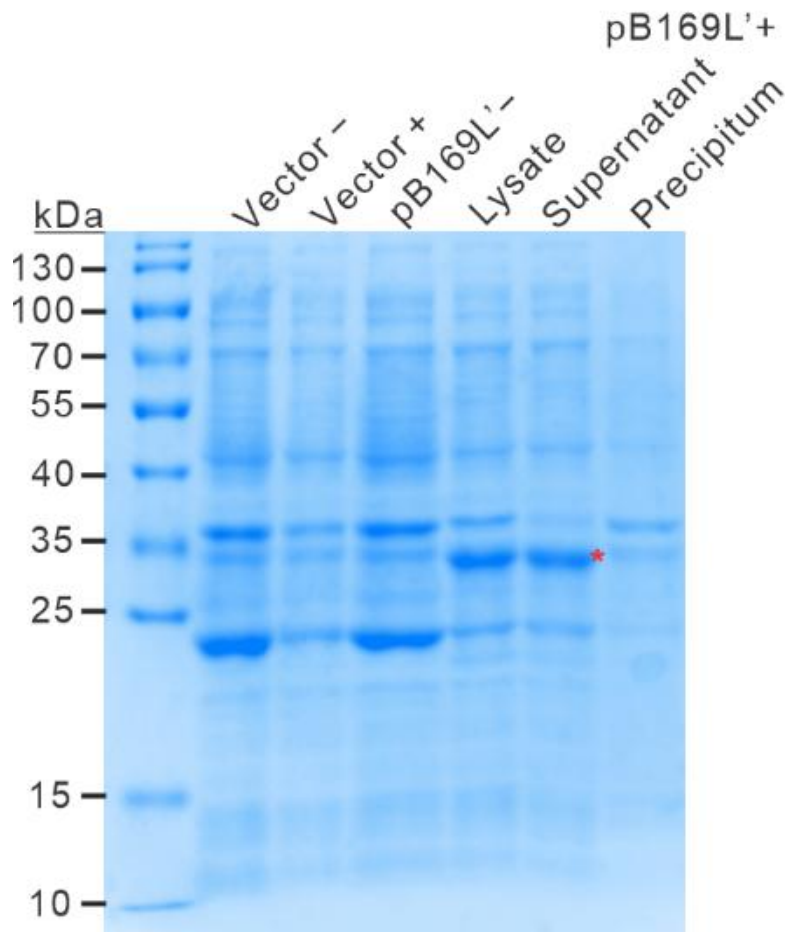

Figure S1. SDS–PAGE analysis of pB169L' expression. pET-28a and pET-28a-pB169L' were separately transformed into Rosetta (DE3). "–" denotes cultivation without IPTG induction, and "+" denotes cultivation with IPTG induction.

Supplementary Table S1. Complete variable region sequences of 1B9

Chain	Type	Nucleotide sequence	Deduced amino acid sequence
VH	IGH	GAGGTAAAGCTGGTGGAGTCTGGAGGAGGCTTGGTACAGCCTGGGGATTCTCTGAGACTCTCCTGTGCAACTTCTGGGTTCACCTTCACTGATTACTACATGAGCTGGGTCCGCCAGCCTCCAGGAAAGGCACTTGAGTGGTTGGGTTTTATTAGAAGCAAAGCTAATGGTTCCACAACATATTATAGTCCATCTGTGAAGGGTCGGTTCACCATCTCCAGAGATAATTCCCAAAGCATCCTCTATCTTCAAATGAACACCCTGAGAGCTGAGGACAGTGCCACTTATTACTGTGCAAGAGATAGCGGGGGAATACTTGACTACTGGGGCCAAGGCACCACTCTCACAGTCTCCTCA	EVKLVESGGGLVQPGDSLRLSCATSGFTFTDYYMSWVRQPPGKALEWLGFIRSKANGSTTYYSPSVKGRFTISRDNSQSILYLQMNTLRAEDSATYYCARDSGGILDYWGQGTTLTVSS
VL	IGκ	GACATTGTGATGTCACAGTCTCCATCCTCCCTAGCTGTGTCAGTTGAAGAGAAGGTTACTGTGAGCTGCAAGTCCAGTCAGAGCCTTTTATATAATGGCAATAAAAAGAACTACTTGGCCTGGTACCAGCAGAAACCTGGGCAGTCTCCTAAACTGCTGATTTACTGGGCATCCACTAGGGAATCTGGGGTCCCTGATCGCTTCACAGGCAGTGGATCTGGGACAGATTTCACTCTCACCATCAGCAGTGTGAAGGCTGAAGACCTGGCAATTTATCACTGTCAGCAATATTATAGGTATCCGTGGACGTTCGGTGGAGGCACCAAGCTGGAAATCAAA	DIVMSQSPSSLAVSVEEKVTVSCKSSQSLLYNGNKKNYLAWYQQKPGQSPKLLIYWASTRESGVPDRFTGSGSGTDFTLTISSVKAEDLAIYHCQQYYRYPWTFGGGTKLEIK
